# Recent updates of eye movement abnormalities in patients with schizophrenia: A scoping review

**DOI:** 10.1111/pcn.13188

**Published:** 2021-01-20

**Authors:** Alexandra Wolf, Kazuo Ueda, Yoji Hirano

**Affiliations:** ^1^ International Research Fellow of Japan Society for the Promotion of Science Fukuoka Japan; ^2^ Department of Human Science, Research Center for Applied Perceptual Science Kyushu University Fukuoka Japan; ^3^ Department of Neuropsychiatry, Graduate School of Medical Sciences Kyushu University Fukuoka Japan

**Keywords:** biomarker, eye‐tracking dysfunction, gaze metrics parameters, schizophrenia, visual information processing

## Abstract

**Aim:**

Although eye‐tracking technology expands beyond capturing eye data just for the sole purpose of ensuring participants maintain their gaze at the presented fixation cross, gaze technology remains of less importance in clinical research. Recently, impairments in visual information encoding processes indexed by novel gaze metrics have been frequently reported in patients with schizophrenia. This work undertakes a scoping review of research on saccadic dysfunctions and exploratory eye movement deficits among patients with schizophrenia. It gathers promising pieces of evidence of eye movement abnormalities in attention‐demanding tasks on the schizophrenia spectrum that have mounted in recent years and their outcomes as potential biological markers.

**Methods:**

The protocol was drafted based on PRISMA for scoping review guidelines. Electronic databases were systematically searched to identify articles published between 2010 and 2020 that examined visual processing in patients with schizophrenia and reported eye movement characteristics as potential biomarkers for this mental illness.

**Results:**

The use of modern eye‐tracking instrumentation has been reported by numerous neuroscientific studies to successfully and non‐invasively improve the detection of visual information processing impairments among the screened population at risk of and identified with schizophrenia.

**Conclusions:**

Eye‐tracking technology has the potential to contribute to the process of early intervention and more apparent separation of the diagnostic entities, being put together by the syndrome‐based approach to the diagnosis of schizophrenia. However, context‐processing paradigms should be conducted and reported in equally accessible publications to build comprehensive models.

*‘Nam ut imago est animi voltus sic indices oculi (…)’*

Cicero, Orator

## Uncovering the evidence: The eye and the brain

Given that the retina develops from the same tissue as the brain, it is the only part of the central nervous system that can be seen with the naked eye in its natural state in living organisms. Therefore, it should not be a surprise that the ability to mirror the brain's integrity of sensory function and its structure has crowned the eyes with the sobriquet of windows to the mind. Although philosophers defined the eyes as the interpreters of one's mind long before neuroscientists, more recently, it has been scientifically confirmed (through vastly improving retinal imagining techniques) that several well‐defined neurodegenerative conditions, as well as psychiatric disorders, find their manifestations in the detailed structures of the human eye.^1^, ^2^ Patients with schizophrenia (SZ) possess an anomalous eye structure; thus, specific retinal findings, such as thickness mapping of retinal layers,^3^ reduced retinal axon layer, macula thickness and volume measurements, in patients with schizophrenia^4^ can serve as structural biomarkers of neural pathology and disease progression.^5^, ^6^, ^7^, ^8^

Undoubtedly, the eye's microarchitectural information can be used as a valuable structural model to study psychiatric disorders to improve the non‐invasive identification of high‐risk individuals.^9^, ^10^, ^11^ Therefore, structural biomarkers remain an increasingly popular topic in clinical applications. However, severe limitations hamper the everyday use of structural targets as far‐reaching and cost‐effective markers for SZ. Meanwhile, a misperception that SZ research findings related to vision are considered minor casts a shadow over gaze metrics as potential neurophysiological biomarkers interpreting the highly heterogeneous brain in patients with SZ.^12^, ^13^, ^14^, ^15^, ^16^, ^17^

Eye‐tracking devices are characterized by relatively cost‐effective assessments and extraordinary flexible data collection locations that can occur in any comfortable environment, and are not restricted to the surrounding of a hospital or psychiatry clinic. Therefore, an eye‐tracking tool introduces a significant promise of accessibility. Also, in the light of a noticeable shift in focus of research in the SZ spectrum from classical symptomatology to context‐processing impairments^2^, ^18^, ^19^, ^20^, ^21^, ^22^, ^23^ and cognitive remediation for treating cognitive dysfunction,^24^, ^25^, ^26^, ^27^, ^28^ the study of cognitive impairments has become a high‐priority area of interest. Finally, following the line of evidence that vision in SZ does matter,^29^ an intriguing trend to study the visual processing structure by re‐examining paradigms with the inclusion of modern eye‐tracking devices started to emerge.^30^

Eye‐tracking datasets obtained from the unconscious movements of their beholders give powerful insights regarding the information processing patterns, and provide accurate instrumentation to measure cognition (and its deficits) at the same time indicating and predicting disease processes.^1^ Although it is possible to attend covertly to a spatial location,^31^, ^32^ it is more effective to fixate the eyes on what one chooses to attend to. Therefore, the fundamental point of eye behavior reflecting one's attention (and consequently thoughts)^33^, ^34^, ^35^, ^36^, ^37^, ^38^, ^39^, ^40^, ^41^ is attractive as a measurable indicator (biomarker) of SZ, detected through an analysis of gaze metrics.^42^, ^43^, ^44^

To systematically present recent contributions of eye‐tracking‐based methodology in research on SZ, a scoping review has been conducted. A research question, which aimed to draw both clinical and psychological scientific communities’ attention to investigate information processing patterns, was formulated. What is known about eye movement impairments in patients with SZ, and what experimental paradigms disclose their presence? Furthermore, saccadic dysfunctions and exploratory eye movements were highlighted as potential biomarkers for SZ. Finally, we identified an inefficient translation of dynamic experimental paradigms expansion (in vision science research) into clinical research, creating a widening gap between scientific evidence and potential clinical application.

## Vision science as a new practice

*Eye movements reflect the human thought processes; so the observer's thought may be followed to some extent from records of eye movements. It is easy to determine from these records which elements attract the observer's eye (and, consequently, his thought), in what order, and how often*.^45^ (*p. 190)*

In 1879, by merely observing the eyes through a system of mirrors, Louis Javal noted that while reading, the eyes do not sweep smoothly across the page, but rather, make a series of brief stops on individual words. These brief stops became known as fixations, while the rapid eye movements between fixations became known as saccades. Later on, Guy T. Buswell (*How people look at pictures*)^46^ and Alfred Lukyanovich Yarbus (*Eye Movements and Vision*)^45^ pioneered the study of cognitive influences acting on visual exploration, researching the interplay between fixation and interest. Deliberately, we mention these two now‐classic, however, surprisingly excluded from clinical research monographs, to inspire scientists to investigate the influence of task' instruction on visual exploration patterns among clinical populations, a topic yet uncovered by neurocognitive research.

Modern eye‐tracking technology is recognized for its sophisticated mathematical image analysis, with its ability to detect viewers’ pupils and calculate the corneal reflection patterns. By probing one's information processing, which requires a synchronized activity of lower and higher‐order brain structures, gaze can mirror the brain's integrity. It has been reported that patients with psychiatric disorders show significant attention deficits in comparison with healthy controls (HC).^2^, ^47^, ^48^, ^49^, ^50^, ^51^ Eye‐tracking studies may facilitate understanding of the neurobiology of populations with mental disorders, and evaluate mechanisms involved in attention processes.^52^, ^53^, ^54^, ^55^, ^56^ For example, reflexive saccades are considered to be type of cognitive parameter that evaluates attention. Furthermore, to unveil insights regarding the complexity of one's eye movement behavior (to process given information), researchers start to record exploratory scan paths during free‐viewing exploration tasks.^53^, ^57^, ^58^, ^59^, ^60^, ^61^ Clarifying aspects of brain circuitry by illustrating the complex interplay between vision and cognition,^62^ eye movement measurements are actively performed in interdisciplinary laboratories that integrate contributions from psychology, neuroscience, economy, philosophy and computer science. A vast number of efficient and well‐grounded behavioral experimental paradigms have been generated regarding sincerity,^63^ magic‐tricks,^64^, ^65^ judgmental tasks,^66^, ^67^, ^68^, ^69^, ^70^, ^71^ moral dilemma,^72^ reading,^73^, ^74^, ^75^, ^76^ visual search,^77^, ^78^, ^79^ gambling,^80^ daily‐life activities,^81^, ^82^, ^83^, ^84^, ^85^, ^86^ problem‐solving,^39^, ^87^, ^88^, ^89^ learning^90^ and discrete choice in clinical decision‐making.^91^ Vision science becomes actively adopted in neuromarketing, aiming to reveal valuable information about consumers’ decisions, as well as answer the question of how to hack the consumer's internal ‘purchase button’.^92^, ^93^, ^94^, ^95^, ^96^

Despite such immense focus on the gaze within cognitive neuroscience and an active import of eye‐tracking paradigms to other fields, vision science has not resulted in a similar focus in mental illness research.^97^, ^98^ Findings of significant importance show that gaze metrics parameters are abnormal in psychiatric diseases, such as depression, bipolar disorder and SZ. Consequently, we state that cognitively informative behavior paradigms might have useful implications for clinical researchers. Namely, to clarify aspects of disturbed brain circuity and visual impairments that manifest among patients with SZ. Of great importance is the thoroughly reported fact that schizophrenia/schizoaffective disorder (SZ/SA) patients are usually accompanied by a broad spectrum of oculomotor alterations.^99^ Previously, Chapman *et al*. investigated the subjective reports of visual perception in early‐stage SZ individuals, where he reported that patients showed perceptual disturbances, which included impaired visual experiences while showing no symptoms of depressive mood.^100^ This has been the most frequent initial behavioral symptom of SZ appearing over a period of 4 years before the first admission, being later followed by negative symptoms and functional impairment.^101^ Another retrospective survey revealed that most participants experienced distortions (in contrast, shape, color and motion) before being officially diagnosed.^102^ In modern research, several independent research groups unanimously reported that patients with SZ have poor ability to maintain their attention, which in turn led to their overall poor perception.^48^, ^103^, ^104^

Gaze metrics are successfully implemented into paradigms that exercise patients’ abnormalities in motion processing,^105^ contrast sensitivity,^106^, ^107^ surround suppression,^108^, ^109^ perceptual organization,^98^ facial emotion recognition^110^ and color processing.^111^, ^112^ Deeper knowledge regarding visual information processing impairments among patients starts to unveil potential neurophysiological markers and facilitate early diagnosis of mental disorders, such as SZ, characterized by misinterpretation in thinking processes, especially delusions (beliefs not based on reality) and hallucinations (inability to differentiate between what is real and what is not).^42^, ^53^, ^57^, ^113^, ^114^ Recent pieces of evidence highlight patients’ exploratory eye movement measurements to be potential trait‐linked markers of vulnerability to the SZ (single eye‐movement test,^115^, ^116^ multiple eye‐movement tests^42^, ^57^, ^117^, ^118^).

## Objectives

An overall misperception that findings related to gaze metrics are considered minor in SZ research has been previously emphasized.^119^ Consequently, to systematically map the findings, a scoping review was conducted that referred to studies showing the potential value of eye‐tracking technology as a way of analyzing cognitive function – following the underlying hypothesis that fixation tends to follow attention. This review is a preliminary attempt to draw readers’ attention to eye‐tracking technology advantages in clinical studies (e.g. experimental paradigms regarding SZ patients). Furthermore, state‐of‐the‐art articles related to visual processing in patients with SZ, reporting abnormal gaze metrics, were identified, and clinical concepts that implement eye‐tracking technology in visual exploration experimental paradigms were discussed. The next decade of SZ research is likely to witness eye movement‐related measurements (gaze metrics) being actively used as potential biological markers. The necessity to include tasks that incorporate cognitive features to currently exercised experiments has also been highlighted. Such interdisciplinary combinations could be beneficial to patients by finding novel ways for early diagnosis of SZ. Finally, the authors undertook the present study to emphasize the importance of a multifaceted medical research team that includes cognitive and computational neuroscience specialists.

## Methods

Our protocol was drafted using PRISMA guidelines, revised by the authors and laboratory members to solicit additional feedback.^120^ Articles published between 2010 and 2020, reporting attention deficits or abnormal exploratory patterns among patients with SZ, were identified by Mendeley and PubMed searches. To narrow the result of over 1100 journal articles, search terms ‘eye‐tracking patterns’, ‘gaze metrics’ and ‘information processing’ were added (in total 75). Journal articles that reported eye movement measurement tests as potential biomarkers for psychotic disorders were localized and added to the scoping review.^42^, ^57^, ^60^, ^116^, ^117^, ^118^, ^121^, ^122^ Reference lists of all in‐scope‐articles have been additionally screened for relevant publications. To interlock the current trend of eye tracking with SZ research findings, non‐clinical open access articles were included in this scoping review. The research synthesis starts with an introduction of gaze metrics used to study cognition while disclosing cognitive disturbances in psychotic populations. Then, saccadic and scan path dysfunctions are highlighted as potential biological markers of symptoms of SZ. Finally, we call attention to eye movement tests having considerable power to discriminate SZ cases from HC.

## (Eye‐)tracking the cognition

Gaze metrics derived from the eye position data are commonly used to study cognition. For example, fixation duration gives insights regarding the looking time at an object, representing the relative engagement and the time dedicated to processing binding features that belong to an object (information). Fixation duration has been used to gain insights into cognitive control capacity; for example, what we remember,^123^, ^124^ how we perform mental computations,^125^ read,^126^ solve problems,^89^, ^127^, ^128^, ^129^, ^130^, ^131^, ^132^, ^133^ compare specific stimuli and make decisions based on integrated pieces of information.^134^ Eye‐tracking technology also provides knowledge about regions of interest, and therefore answers essential questions of when and how information is being captured and processed during vision‐oriented tests.^92^, ^135^, ^136^, ^137^, ^138^, ^139^, ^140^ Of great interest is the time spent on an image; this gaze metric has been widely covered in the scientific literature and is associated with the visual expression of preference.^141^, ^142^, ^143^, ^144^

In the context of SZ, cognitive factors (increased attention, reflexive saccades suppression, intrusive saccades presence) are studied in experimental tasks; that is, gaze tracking of a moving target (*horizontal pursuit paradigm*) and gaze maintenance on a fixation target presented in the center of the monitor (*fixation stability test*). In contrast, some research questions require analysis of scan paths, rather than quantification of fixation and saccade measures.^60^, ^145^, ^146^, ^147^ Thus, the interest in exercising patients’ visual engagement driven by an individual's preferences, beliefs and intuition started to emerge.^148^, ^149^, ^150^ Although free‐viewing tests are currently applied to objectively illustrate patient's visual exploration paths, a minor amount of clinical paradigms implement higher‐order cognitive components that relate to abstract thinking, perspective‐taking (more commonly referred to as the theory of mind) and decision‐making. To investigate the interplay between cognition and vision, more complex information processing paradigms that reveal spontaneous, task‐oriented gaze patterns are required.^33^, ^112^, ^151^, ^152^, ^153^, ^154^, ^155^, ^156^, ^157^, ^158^

Furthermore, throughout the years, human eye behavior has been exercised with a vast amount of different types of stimuli (i.e. geometrical figures, paintings, moral picture database, computerized faces, photographs of human real faces, naturalistic food images) applied in various context‐processing paradigms.^32^, ^35^, ^41^ In the context of SZ, patients have been reported to avoid looking at salient regions of the face.^159^ Such a restricted visual scan path is associated with lower emotion recognition accuracy.^159^ However, these findings relate to ‘bottom‐up’ sensory processing, where ‘bottom‐up’ atypicalities may result from atypical eye‐gaze patterns or deficits of attentional modulation of neural activity. Further studies are required to investigate sensory processing, in the context of visual scan paths to elucidate whether an atypical function of sensory regions can genuinely be considered a ‘bottom‐up’ deficit in SZ. In particular, the use of visual illusions stimuli may demonstrate how the brain uses highly adaptive mechanisms that allow top‐down perceptual mechanisms. In SZ, this top‐down modulation of social perception has been reported as abnormal (patients demonstrate abnormally strong influences of prior expectations).^159^ The opposite appeared to be true among autism patients, characterized by abnormally weak top‐down modulation.^159^, ^160^ The addition of high‐ and low‐level illusion stimuli to future cross‐diagnostic paradigms may contribute to the current knowledge of what symptomologies in SZ have in common, and clarify the understanding of visual distortion symptoms rather than hallucinations.

## Cognitive disturbances as crucial features of schizophrenia

For the reason that almost every area of the brain plays a role related to the ocular motor control system, gaze metrics have been applied to fields of exploration, such as social behavior, design, marketing and consumer sciences, becoming essential for neuroscientists who look at decision‐making through the lens of information processing.^143^, ^161^, ^162^ Although suited to today's challenges, eye‐tracking technology was cut out from clinical studies, mostly by event‐related potential and oscillatory measures, as a way to discover the mechanisms that underlie patients’ behavior, as well as a tool supporting mental illnesses diagnosis. Furthermore, the dynamic expansion of cognitively informative paradigms in vision science is inefficiently translated into psychiatry, widening the gap between scientific evidence and potential application. At the same time, cognitive deficits are repeatedly named to be key features of SZ. It has been scientifically reported that a large proportion of individuals at clinical high risk has poor ability to maintain their attention.^163^, ^164^, ^165^ In recent studies, valuable shreds of evidence regarding cognitive deficits have been confirmed to be highly prevalent in SZ,^166^, ^167^, ^168^ and present even before the onset of the disease.^169^ It has been reported that by the time of the onset of psychosis, where patients experience their first episode of psychosis, attentional impairments are typically present and of moderate severity.^170^ Furthermore, of high importance are studies that report longitudinal persistence of cognitive deficits, thus, suggesting that information processing abnormalities are probably related to the core of the SZ.^171^, ^172^, ^173^, ^174^ In comparison with bipolar disorder or those with drug‐induced psychosis, it has been reported that after the acute psychosis abates, information‐processing deficits persist selectively in SZ patients,^175^ dramatically impoverishing the patient's real‐world functioning.

Notably, most SZ patients and high‐risk states for psychosis individuals demonstrate at least some cognitive impairments, which remain relatively stable within the same patient over time. Therefore, cognitive deficits seem to be unique due to their general consistency in severity and topography across the individual's fluctuating clinical status.^12^, ^24^, ^116^, ^176^

The relatively inexpensive, safe and far‐reaching eye movement technology can mirror cognitive deficits in psychotic patients. Therefore, it is necessary to undertake detailed investigations of visual information processing patterns among psychiatric disorder patients, especially those with SZ spectrum disorder.^29^, ^177^, ^178^ Recent essential signs of progress, such as the use of integrated eye movement measurements to distinguish patients with SZ from HC, support the statement that the *interpretations of one's attention* in the form of eye movement measurements should be actively studied in SZ research.^1^, ^42^, ^47^, ^54^, ^62^, ^98^, ^179^, ^180^, ^181^, ^182^ Finally, studies of vision in SZ have an enormous potential to identify sensitive probes of neural functioning that can be used as neurophysiological biomarkers.

The next part of our scoping review attempts to describe abnormalities in eye movements and visual information processing among patients with SZ, providing a disorder‐specific marker value. Furthermore, currently implemented clinical *viewing tests* (i.e. *free viewing*, *horizontal pursuit*, *fixation stability*) distinguishing patients with SZ from the HC have been outlined. Finally, we underlined the necessity to incorporate cognitive features to generally exercised experimental paradigms, as extracting task‐relevant information from an external input and acting accordingly to internal preferences and/or given experimental tasks requires a balance between receptor structures (in order to, e.g., visually scan the environment) and the central circuit (to process and interpret the incoming information).^183^ Thus, to understand the real‐life interplay between cognition and eye behavior, clinical researchers may consider introducing ecologically valid situations into experimental paradigms, monitoring the gaze during monitor display and social communication mode. Gained knowledge of how information is being extracted and further manipulated may serve as a background for future comparisons across various psychiatric disorders.

## Clinical concepts that implement eye‐tracking technology

In 1903, the first study of eye movements in SZ (dementia praecox patients at that time) had been published, in which Kraepelin reported an incomplete perception of very briefly exposed stimuli. In 1908, psychiatrist Allen Diefendorf and experimental psychologist Raymond Dodge collaborated to study ocular motor function in psychiatric patients.^184^ To capitalize on over‐learned visual behavior, they chose to study smooth pursuit and reflexive saccades that avoid any confounding effects of tasks that could be ‘too complicated’ or have ‘unusual demands’ for patients (hence, any found deficits would suggest disease‐related dysfunction in a potentially informative neural system). Diefendorf and Dodge's landmark study of eye movements in psychiatric patients laid the foundation for further investigations. However, with Bleuler's report of being unable to replicate Kraepelin's findings (in *Dementia Praecox or the Group of Schizophrenias* 1911), research on visual processing in SZ diminished. It peaked during the ‘cognitive revolution’ (in the 1950s and 1960s), when the number of cognition‐based experiments increased and the interest in oculomotor disturbances associated with psychoses re‐emerged.^47^, ^180^, ^181^, ^185^, ^186^

The independent rediscovery by Holzman *et al*. revealed an eye‐tracking dysfunction (or smooth pursuit eye movement impairment) not only in patients with SZ, but also in almost half of their clinically unaffected first‐degree biological relatives.^180^ This research team made a great contribution to the field by: (i) replicating and extending previous findings of Diefendorf and Dodge; and (ii) directing the focus of SZ research primarily on motion perception tasks. Afterwards, a vast number of studies reported biological motion sensitivity delineating patients with SZ from those with bipolar disorder (for a comprehensive review, refer to Okruszek *et al*.^187^). Until now, albeit other factors that have been scientifically reported to be abnormal in patients with SZ, eye‐tracking dysfunction remains above all as a trait abnormality limited to the risk for SZ.^15^, ^105^, ^116^, ^188^, ^189^, ^190^, ^191^, ^192^

The smooth pursuit eye‐movement serves to keep swinging in a harmonic motion object foveated in the field of one's vision.^193^ Although the smooth pursuit task provides information about the patient's voluntary behavior, in the sense that the observer can decide whether or not to track a moving stimulus, it basically relies on visual cues indicating motion of the visual field. Hence, aiming to investigate the sensory system coordinating movement (prioritized information search) with balance (stabilizing the eyes on a stationary figure reflecting cognitive processes), researchers start to record eye movements during *free‐viewing visual exploration tasks* and unfold key findings (i.e. abnormal gaze behavior) in SZ patients. Finally, more than 100 years after Kraepelin's work, evidence of saccadic and scanning patterns dysfunctions among SZ patients start to be highlighted in scientific publications. Here, we aim to outline these abnormalities as potential biological markers of symptoms of SZ, as well as highlight eye movement tests discriminating SZ cases from HC.

## Saccadic dysfunctions among patients with schizophrenia

Over the past three decades, the majority of studies among psychiatric patient groups focused on saccadic performance. Much of the impetus for such focus came from the fact that saccades provide accessible means of investigating psychomotor functioning, higher‐order cognitive processes and their underlying neural mechanisms, remaining non‐invasive at the same time. Furthermore, saccadic eye movements can be measured reliably and precisely. As a result, a considerable body of knowledge regarding the neurophysiology of the saccadic eye movement subsystem has been shaped, suggesting that saccadic eye movements in psychiatric groups provide a ‘window into the brain’ of affected individuals.

Within 30 years after Holzman's studies, the crucial finding of eye‐tracking dysfunction in SZ has been consistently reported in an extensive number of replications,^180^, ^194^, ^195^, ^196^, ^197^, ^198^, ^199^, ^200^, ^201^ where frequent small saccades were pointed out to be the distractors of eye behavior. Oculomotor control in psychiatric populations has been studied with a range of saccade paradigms, such as *prosaccades* (saccades to target sequences), *antisaccades* (saccades away from targets) and *saccades to remembered targets*, demanding an increase of attention.^48^, ^55^, ^202^, ^203^, ^204^, ^205^ Orienting toward a stimulus is reflexive, and moving one's eyes to the opposite location requires inhibitory control and maintenance of the task's instruction. Poor inhibition has been linked with low impulse control, agitation, excitement and hostility, as well as impairment in the dorsolateral prefrontal cortex.^206^, ^207^ Thus, the antisaccade task (used to explore one's inhibitory control) generates the most frequently observed volitional saccade abnormality among SZ patients.^208^ Combined with an increased rate of errors reflecting impairments in the suppression of reflexive saccades, antisaccades represent one of the most replicable findings in SZ research,^209^ drawing a line between HC and SZ.

Of course, regarding the literature on gaze metrics in psychiatric research, it has been strongly underlined that a single task may not serve as a reliable diagnostic tool. A combination of gaze metrics obtained by multiple tasks, however, may increase the classification accuracy to distinguish patients from HC, as well as characterize particular clinical dimensions of SZ.^42^, ^117^

A gripping saccade‐oriented study, recently reported by Obyedkov *et al*., included patients divided into three subgroups.^54^ First with predominantly negative symptoms, second with predominantly positive symptoms and third with predominantly disorganization symptoms. Horizontal eye movements, peak velocity, latency and accuracy were recorded in all saccade‐oriented tasks (i.e. *prosaccade*, *antisaccade* and *predictive saccade tasks*). Additionally, in the antisaccade task, the error rates were measured. In agreement with previous reports, all SZ patients performed worse than HC across all tasks.^210^, ^211^ The results of Obyedkov *et al*. revealed that all three groups of SZ patients made a higher rate of errors (in the antisaccade task) than HC; which reflects a failure of response suppression. Notably, patients with disorganization symptoms made more errors in the antisaccade task than the two other subgroups, which in turn confirmed that disorganization symptoms are associated with (i) attentional dysfunction and (ii) failure to suppress inappropriate responses.^209^ Furthermore, patients with negative symptoms were characterized with different oculomotor parameters than in other subgroups. This result has found consistency with the available literature regarding motor, cognitive and neuropathological differences in patients with prominent negative symptoms when compared with patients without negative symptoms (for involvement of the prefrontal cortex in pathological processes in patients with predominantly negative symptoms that may result in the abnormalities of saccadic eye movements see Obyedkov *et al*.^54^). Furthermore, latencies compared with accuracies were reported to be more closely associated with negative symptoms of SZ. Notably, prolonged latencies among patients with positive symptoms appeared in both predictive and reflexive tasks, which in turn provided valuable feedback that latencies might serve as markers of negative symptoms of SZ.

The provided reports have raised the possibility that abnormal saccadic eye movements might be an alternative manifestation of SZ.^54^, ^197^, ^212^ Furthermore, the necessity to study clinically unaffected relatives of SZ patients has been emphasized, as an elevated rate of eye‐tracking dysfunctions in clinically unaffected relatives and clinically discordant co‐twins has been frequently reported.^48^, ^54^ To date, only a small number of studies have investigated performance on saccadic tasks in high‐risk clinical populations. It is essential to mention that the group of clinically unaffected relatives has been reported to differ significantly from HC. In particular, the performance on the antisaccade task among high‐risk clinical participants has been reported to be close to that of SZ patients. Therefore, saccadic measures may have the potential to become biomarkers in translational medicine of psychosis.^54^

## Abnormal exploratory eye movements among patients with SZ

By recording gaze parameters, such as eye fixations (their number and sequence), scanning path (total and mean length) and responsive search score (RSS), eye movement patterns in patients with SZ are reported to be different from those observed among mood disorder patients and HC (^115^, ^116^ reported by Morita *et al*.^42^). Previously, pioneering exploratory eye movement experiments, conducted with HC, have shown that observers’ fixations reflect their cognitive processes.^45^, ^145^, ^213^ Following the knowledge that the choice of one's visual fixation is influenced by the aspects of the target, as well as peripheral characteristics of the stimulus, a significant number of studies inspected SZ patients while freely viewing stationery ‘S’ shaped figures (happening to reflect visuocognitive dysfunction).^113^, ^121^, ^193^, ^214^, ^215^, ^216^, ^217^

By showing a horizontal ‘S’ shaped figure to chronic SZ patients and their parents, and recording their exploratory eye‐movements, Moriya *et al*. found that SZ patients made fewer movements in comparison with HC.^218^ Also, the range of eye movements among SZ patients was narrower. Interestingly, their parents’ results were reported to lie mid‐way between SZ patients and HC. Moriya *et al*. concluded that exploratory eye movement dysfunction reflects the trait of Sz.^216^ In 1990, Kojima *et al*. introduced participants to a figure, which was slightly different from the initially shown ‘S’ shaped target.^214^ The research team recorded exploratory eye movements (EEMs) of the original and slightly changed targets, and found that SZ patients (chronic, acute and those in remission) made fewer eye movements in response to the verbal stimuli in the comparison test (‘*Are there any other differences?*’). Researchers stated that the RSS represents the visual behavior that anticipates and confirms the difference in both ‘S’ shaped figures. Furthermore, it has been reported that RSS correlates to symptoms associated with SZ (blunted affect, emotional withdrawal and avolition/apathy).^115^, ^214^ In 1998, Matsushima *et al*. reported RSS and the number of eye fixations (NEFs) to differentiate between patients with and without SZ, with a sensitivity of 76.7% and specificity of 81.4%.^193^ The following formula was used: D = 10.265 – (0.065 × NEF + 0.871 × RSS), whereby substituting RSS and NEF values for each participant, a positive result for D indicated SZ. The validity of the mentioned discriminant function was confirmed in 2001, when exploratory eye movements made on horizontal ‘S’ shaped figures, and a slightly different ‘S’ shaped figure displayed alternately, were recorded in seven World Health Organization collaborative centers.^121^ The RSS was confirmed by Kojima *et al*. in 2001 as an exploratory eye movement parameter, which detects SZ irrespective of culture and race (i.e. patients with SZ were discriminated from depressed patients and HC with a sensitivity of 89.0% and specificity of 86.7%).^121^

In 2009, Suzuki *et al*. performed a discriminant analysis between SZ and non‐SZ participants, using only EEM data. Although all parameters (i.e. NEFs, total eye scanning length [TESL], mean eye scanning length and RSS) were reported to differ between SZ and non‐SZ participants significantly, the authors performed a discriminant analysis using two parameters, namely: RSS and TESL.^217^ Utilizing the following discriminant formula: D = 4.100–(0.001 × TESL + 0.332 × RSS), 184 of the 251 clinically diagnosed SZ patients were identified as true positives (with a sensitivity of 73.3%). Whereas 308 of the 389 clinically diagnosed non‐SZ participants (250 HC, 111 mood disorder patients and 28 patients with neurotic disorder) were identified as true negatives (with a specificity of 79.2%).^217^ Hence, Suzuki *et al*. considered EEM parameters as being useful for clinical diagnosis of SZ.^217^ However, as 184 of the 251 clinically diagnosed SZ patients were identified as having SZ, the remaining 67 SZ patients were wrongly discriminated as being non‐SZ. Therefore, in a follow‐up study in 2012, Suzuki *et al*. compared demographic and symptomatic characteristics of identified SZ patients with SZ patients identified as not having SZ.^113^ Suzuki *et al*. concluded that EEM parameters might potentially detect SZ patients with severe symptoms related to excitement/hostility, negative symptoms and disorganization. However, to provide stronger evidence for an exploratory eye movement test simplifying the heterogeneity of SZ patients, further experiments (involving more participants) need to be performed.^113^

The listed investigations showed that exploratory eye movement dysfunction appears to be specific to SZ.^115^, ^121^, ^193^, ^217^, ^219^, ^220^, ^221^ Of course, as SZ is considered to be a cluster of several biologically different conditions, the clinical community cannot expect to cover all aspects by a single marker,^193^ following an important note made by Matsushima *et al*., ‘*(…) exploratory eye movements alone cannot be used to pick up all schizophrenics’*. (p. 294).^193^ However, investigating the correlation between exploratory eye movements (especially RSS) and structural characteristics of the brain (e.g. gray matter cytoarchitectural characteristics) may contribute to the classification accuracy in distinguishing SZ patients from HC. Also, such an interdisciplinary approach may help to characterize particular clinical dimensions of SZ.^222^

As RSS is an integrated measure of the ability for (i) discrimination, (ii) sustained and selective attention, (iii) perception, and (iv) working memory, any impairment related to SZ would reflect by significantly reducing this particular score. At the same time, the available literature suggests that abnormality in brain structure is one of the most critical pathological substrates that underlie SZ. In 2005, Tsunoda *et al*., combined these two facts and conducted a pioneering study, exploring the relationship between EEM and the brain's morphology.^223^ The findings concluded that among schizophrenia spectrum patients, RSS is significantly correlated with gray matter density in the right frontal eye field and right inferior frontal region.^223^

Following the study of Tsunoda *et al*., Qiu *et al*. reported that patients with SZ showed lower RSS (in comparison with HC).^221^ Furthermore, their crucial finding showed that SZ patients’ decreased RSS is significantly associated with gray matter density reduction (in the occipito‐temporal‐frontal circuitry, involved in processing visual information and the control of eye movements). These findings underline that lower gray matter density is related to the EEM disturbances among SZ patients. Therefore, the impaired RSS can be used as a biomarker (phenotype and vulnerability) for SZ.^219^, ^221^ In 2018, Qiu *et al*. reported that hallucination severity among SZ patients was significantly negatively correlated with both RSS and gray matter volume.^224^ Importantly, in HC, no significant association between RSS and the gray matter volume of any brain regions could be shown.^224^ Investigating the correlations between RSS of EEM, hallucination severity and gray matter volume supported the hypothesis of Qiu *et al*. regarding the RSS being not only a potential biomarker of SZ, but also predicting hallucination severity.^221^, ^224^

Overall, brain volume alterations, such as gray and white matter volume loss, enlarged ventricles, and change in the volume of the anterior cingulate cortex, insula and thalamus, have been identified in SZ patients.^221^, ^224^, ^225^, ^226^ Notably, cognitive impairments that determine the quality of life and social functioning among patients with SZ have been reported to be related to brain structures.^222^, ^226^, ^227^, ^228^, ^229^, ^230^ However, of great importance is the fact that brain structure shows maturational changes;^231^ for example, gray matter cortical thickness decreases as a function of age. Age‐related changes are observed for white matter, with an age‐related increase in fractional anisotropy due to the myelination of white matter. In conclusion, brain imaging findings report an effect of brain maturation on diagnostic markers.^231^ The strong correlation between age and brain measures may limit the ability to detect distinct structure–function associations. In future studies, large samples with a small age range should be of interest (i.e. reducing the influence of maturation by restricting age), providing better structure–function estimates.^231^

Based on these findings, several independent research groups underlined the necessity to incorporate vision research into brain imaging studies; to investigate the relationship between eye movements, and structural and functional brain abnormalities in cognitive subgroups of SZ.^225^, ^226^, ^227^, ^232^, ^233^ Furthermore, to show eye movement abnormalities among SZ patients and their relationship with patients’ social/intellectual functioning,^182^, ^234^ researchers actively studied the association between eye movement abnormalities and (i) work hours (obtained through the Social Activity Assessment),^235^ and (ii) the Wechsler Adult Intelligence Scale.^44^, ^182^, ^230^ However, further research questions regarding the relevance between exploratory eye movements and everyday life functioning (e.g. Utena's Brief Objective Measures, developed to assess psychophysiological functions proximal to real‐world functioning among individuals with psychiatric disorders, including SZ^236^) need to be studied in longitudinal investigations with large samples of a small age range (reducing the influence of maturational changes).^44^, ^231^

Scan paths can be a valuable addition to eye movement trait markers, drawing a line between SZ patients and HC. Gazing at only one section of presented stimulus, patients with SZ were reported to tend to shift their fixation less frequently than HC, who view pictures widely.^112^, ^237^ Multiple research teams, who used free‐viewing tasks and re‐examined eye movement patterns, confirmed SZ patients’ scanning patterns to be abnormal, and their regions of interest to be atypical.^57^, ^113^, ^117^, ^121^, ^217^, ^238^ The free‐viewing experimental construct is considered beneficial, as it provides information about the participant's visual scanning behavior, in a sense that the observer may freely redirect the point of fixation according to subjective preferences, goals and beliefs (facts, opinions, uncertainties). Hence, performing free‐viewing paradigms may be useful to investigate visual scanning patterns and understand the patient's actions in a real‐life functioning context.^53^, ^112^, ^237^

Following well‐established reports of face‐scanning patterns among HC (for a description of cyclic fixation behavior while viewing pictures of faces refer to Yarbus^45^), some clinical studies have shown that SZ patients do not pay attention to remarkable facial characteristics while viewing pictures of recognizable and not recognizable faces.^239^, ^240^, ^241^, ^242^, ^243^, ^244^, ^245^ To summarize, patients with SZ tend to have significantly fewer fixation points in comparison with HC. In addition, unlike HC, they show inattention of salient facial features (i.e. eyes, nose and mouth).^159^ These results suggest that atypical visual scanning might impair complex object cognition, such as face perception.

Therefore, another example are studies that focus on the SZ patients’ scanning behavior during facial recognition and facial affect recognition tasks. In 1997, Streit *et al*. reported that patients with SZ show atypical scan paths during the facial affect recognition test.^239^ In two more recent studies, where the relationship between facial affect recognition and scan path features in patients with SZ has been examined, Loughland *et al*. observed patients with SZ showing restricted (characterized with fewer fixations) and abnormal (inattention to significant silent features) scanning styles.^240^, ^241^ Furthermore, both studies provided another valuable insight; namely, that first‐degree relatives of patients with SZ show restricted exploratory patterns, which again pointed out an earlier conviction that examining not only patients with SZ, but also their relatives, is of great necessity.

To break the pattern of face recognition investigation,^242^, ^243^, ^244^, ^245^ Sprenger *et al*.^60^ conducted a multisite study to investigate visual exploration patterns in patients with SZ. The research group used six situational pictures, containing cognitive and emotional elements. The authors compared changes in exploration behavior, cluster analyses, attentional landscapes and analyses of scan path similarities between two groups (HC and SZ). Patients with SZ showed longer fixation times and fewer fixation shifts than HC. They also seemed to generally use a more focal processing mode with longer fixations on distinct features in the center of a scene, in contrast to a more ambient processing of context information (seen in HC). Furthermore, despite visual alterations, patients with SZ appeared to be able to adapt their visual exploration strategies to changes in cognitive complexity, physical properties and emotional strain (as HC did). Interestingly, the study showed that cognitive and emotional elements of the presented stimulus significantly affected the visual exploration of both groups.

Another study stressed the possible existence of a relationship between exploratory scanning patterns and the symptomatic dimensions (positive/negative) of SZ. In a free‐viewing task, Gaebel *et al*. used pictures showing an interpersonal communication situation within the context of a proverb.^147^ In this particular study, patients who manifested more negative symptoms were reported to use a staring‐scanning method, whereas patients with more positive symptoms disclosed an extensive‐scanning method.

## Visual information processing abnormalities in schizophrenia may have a disorder‐specific‐marker value

Indeed, one may say that atypical visual scanning patterns have been shown not only in SZ patients, but in individuals with other psychiatric disorders, including social phobia,^246^, ^247^ bipolar affective disorder,^248^ attention‐deficit/hyperactivity disorder,^131^, ^249^, ^250^ generalized anxiety disorder, and with persecutory delusions,^251^ Asperger syndrome^252^ and autism (e.g. superficially normal attentional preference for social information in adults with autism spectrum disorders [ASD]^253^, ^254^, ^255^). However, the abnormalities found in the aforementioned psychiatric disorders differ from those found in individuals with SZ. Brakemeier *et al*. reported that sensorimotor and cognitive processing impairments are relatively specific to those with a history of psychosis.^122^

**Fig. 1:**
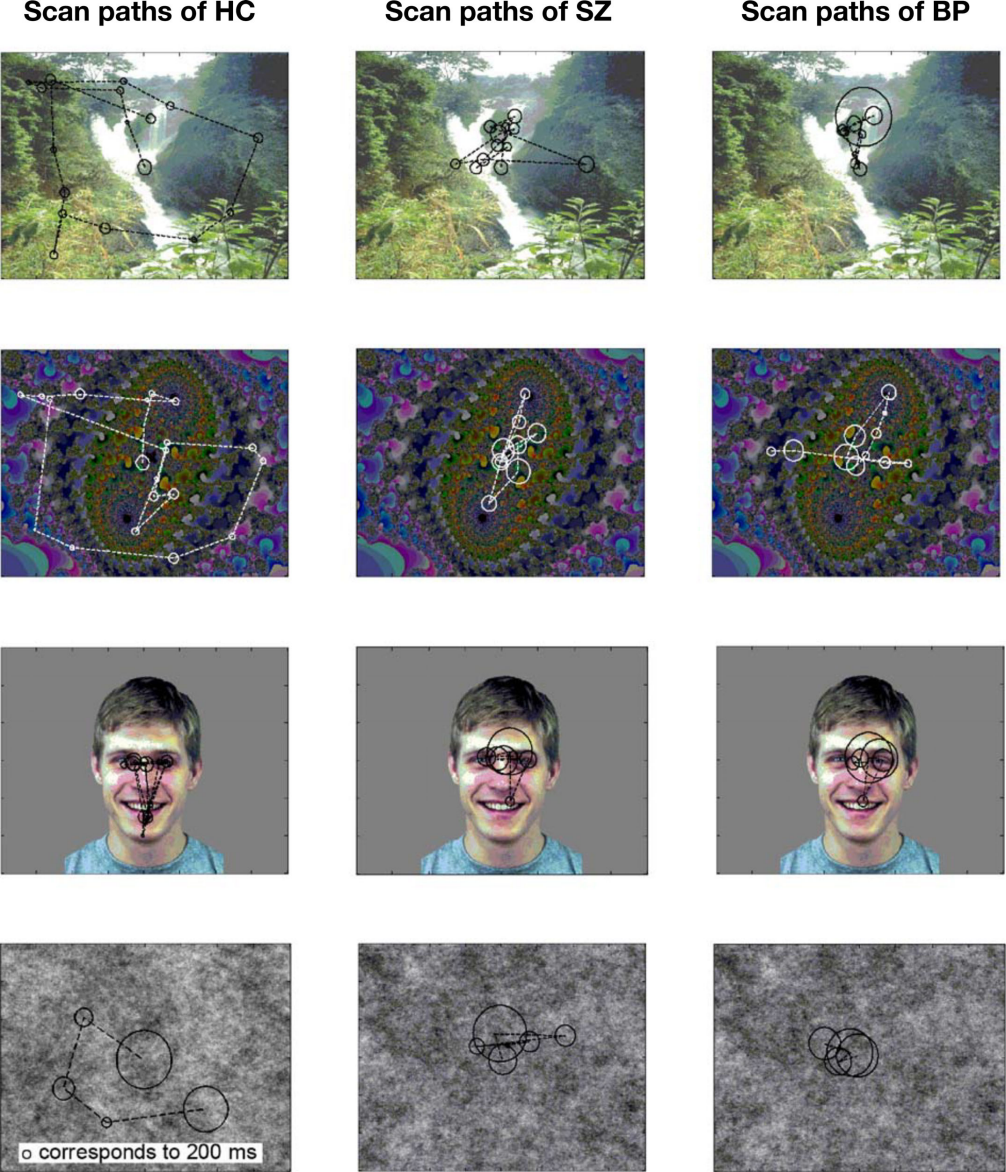
Representative visual scan paths to each stimulus type for one participant from each of the three groups (healthy control, schizophrenia and bipolar disorder). Reproduced from Bestelmayer *et al*.,^248^ with permission.

With an advantage, studies of eye movements in SZ have investigated visual scanning pattern paradigms that instructed the participants to identify the displayed emotional expression. The study of Bestelmayer *et al*., where the participants were asked to view presented images freely, replicated the well‐established finding of restricted visual scanning of faces in SZ.^248^ The most remarkable result to emerge from the data was that a restricted visual scanning effect was present in all types of used stimuli (landscape, fractal and noise stimulus). Previous findings by Loughland *et al*., who suggested individuals with SZ were more impaired on facial stimuli compared with images without social content, were not supported in the study.^240^, ^241^ Instead, the visual scanning abnormalities in patients with SZ are suggested to reflect a global scanning impairment (Fig. 1).

Furthermore, the sample of patients with bipolar disorder showed a more homogeneous spatial distribution of scanning of social stimuli compared with the sample of patients with SZ (the SZ within‐group variation was reported to be significantly different from the bipolar disorder within‐group variation on all but face images). As previously highlighted, the investigation of scanning patterns may expose differences within the psychoses not necessarily detectable by temporal measures alone.^248^ Therefore, further investigations are required to determine the value of spatial measures as biological markers for stratifying the functional psychoses.

Notably, several imaging studies have reported an overlap of the impaired brain circuit among SZ patients with that of individuals with autism.^256^, ^257^, ^258^, ^259^, ^260^ As autism spectrum disorder and SZ patients (without positive symptoms) share common cognitive symptoms, to differentiate them and state a precise diagnosis has been reported to be arduous. This has led Fukushima to examine voluntary control of saccadic and smooth pursuit eye movements among adults with SZ and autism spectrum disorder, and compare the results with the performance of a typically developed (healthy) group.^261^ Although 38% of the autism spectrum disorder participants were reported to have higher error rates in the antisaccade task and expected gains in horizontal sinusoidal smooth pursuit, approximately 70% of patients with SZ showed abnormalities in the antisaccade task. Furthermore, in the smooth pursuit task, 70% of adults with SZ showed a lower gain than HC. Abnormalities in eye movement tasks have been reported to be stronger among patients with SZ rather than ASD.^261^ In conclusion, eye‐tracking evidence was reported to offer insight into circuit‐level alterations, which have become promising biomarkers of autism and SZ disorder.

**Fig. 2:**
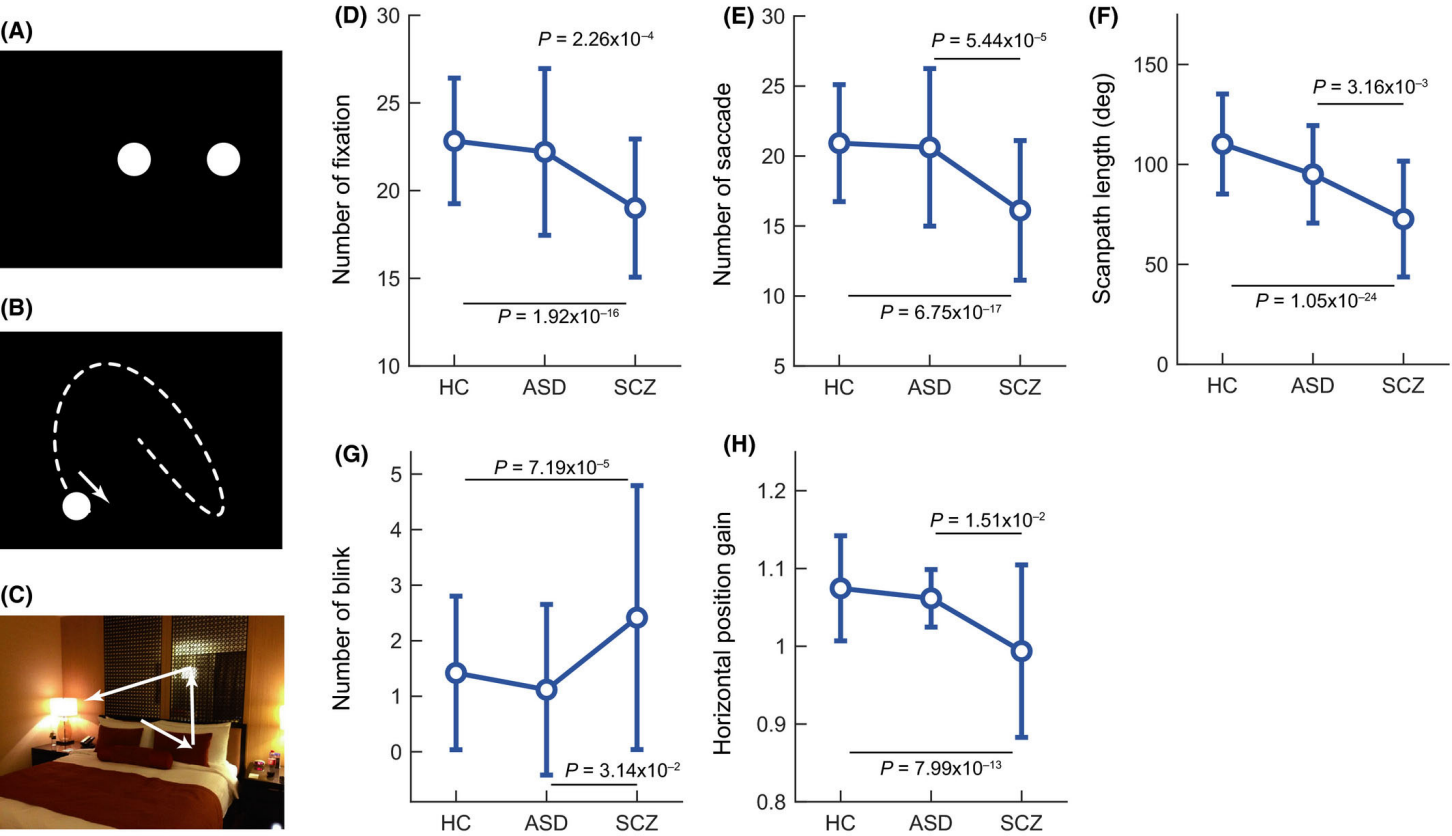
Schematic diagrams of stimulus paradigms: (a) fixation task, (b) smooth pursuit task, (c) free viewing task and comparisons among groups in five characteristics – (d) number of fixations, (e) number of saccades, (f) scan path length and (g) number of blinks – in the free viewing eye movements and the (h) horizontal position gain in the smooth pursuit eye movements. ASD, autism spectrum disorder; HC, healthy control; SCZ, schizophrenia. From Shiino *et al*.,^262^ with permission.

Following Fukushima's conclusions that eye‐tracking deficits have been localized in individuals with SZ rather than ASD,^261^ a more recent study was conducted by Shiino *et al*. that aimed to understand the similarities and differences in eye movement abnormalities across patients diagnosed with SZ and those with ASD.^262^ Out of 75 collected eye movement characteristics, the research group successfully pointed out five characteristics where individuals with SZ showed significant differences from the individuals with ASD (4 during the free viewing task: number of fixations; number of saccades; scan‐path length; number of blinks; and 1 during the smooth pursuit eye movements task: horizontal position gain; Fig. 2). Visual information processing abnormalities have been confirmed to have a disorder‐specific marker value in SZ patients. Further gaze measuring paradigms may uncover global similarities in eye behavior abnormality patterns among various groups of patients, as well as state differences between them. However, to establish such comparison across various psychiatric disorders, intensive investigations are required.

## Modern clinical concepts that implement viewing tests

More and more modern studies, with the use of state‐of‐the‐art eye trackers, corroborate the fact that a reduced integration of visual information contributes to altered visual experiences in patients with SZ.^1^, ^48^, ^60^, ^104^ Importantly, perception deficits seem to persist exclusively (selectively) in SZ. Therefore, in recent years, some independent research groups have started to actively record eye movements to differentiate patients with SZ from HC.^23^, ^42^, ^54^, ^117^, ^219^, ^238^, ^263^, ^264^, ^265^ Such an approach marks a new beginning of promising continuous years of research on visual disturbances in SZ patients, with eye‐tracking technology revolutionizing mental health screening, even with the use of one's smartphone.^266^, ^267^, ^268^ Pioneering studies, which significantly contributed to the development of discriminant analysis that incorporates eye movement parameters to distinguish SZ cases from HC, are presented in Table 1. Whereas, modern clinical concepts, published between 2010 and 2020, which implement viewing tests that significantly differ between participants with and without SZ, are chronologically described below.

**Table 1:** List of pioneering and modern studies distinguishing schizophrenia patients from healthy controls by eye‐movement parameters measured across *(i)* tests with oscillating targets, *(ii)* tests with table targets and *(iii)* a combination of eye‐movement tests

Author(s)	No. participants	Suggested eye‐movement discriminator(s)	Stimuli	Test	Summary of key findings
*(i) Oscillating stimulus*					
Holzman *et al*., 1973^179^	25 psychotic patients (18 SZ) 8 psychiatric controls 33 HC	SPEM	Oscillating pendulum	Smooth pursuit	SZ patients show smooth pursuit eye‐tracking patterns that differ strikingly from the generally smooth eye‐tracking observed in HC and in non‐SZ probands. These deviations refer to oculomotor involvement, having a critical relevance for perceptual dysfunction in SZ.
Holzman *et al*., 1974^180^	69 SZ 6 schizoaffective disorders 9 manic‐depressives 19 non‐psychotic patients 34 relatives of SZ 19 relatives of non‐SZ 72 HC	SPEM	Oscillating pendulum	Smooth pursuit	Smooth‐pursuit eye movement test disclosed a striking association between deviant eye‐tracking and clinically diagnosed SZ. Disruptions of smooth pursuit eye movements (SPEM abnormality) were found in 52% of recent and 86% of chronic SZ patients as well as 44% of their first‐degree relatives. SZ probands and their biological family members have a deficit in an accurate smooth pursuit. The eye‐tracking dysfunction may represent a genetic marker, highly useful for studying the transmission of a vulnerability to SZ.
Holzman *et al*., 1984^195^	80 SZ 46 manic‐depressives 59 parents of SZ patients 48 HC	SPEM	Oscillating pendulum (yellow cross)	Smooth pursuit	Eye‐tracking dysfunctions are reliably found in 50–85% of SZ patients, about 40% of manic‐depressive patients, and about 8% of the HC. Deviant eye‐tracking in pursuit of eye‐movements reported among family members of SZ. Parental eye‐movement dysfunctions are significantly related to the patient's diagnosis. In the absence of other CNS diseases, ETD represents a familial marker of vulnerability to SZ.
*(ii) Stable stimulus*					
Moriya *et al*., 1972^218^	24 chronic SZ 20 HC	*‐NR‐* ^†^	Human figures and ‘S’ shaped figures	Free viewing	Chronic SZ patients show atypical scanning patterns (fewer fixations and a narrower range of eye‐movements in chronic SZ in comparison to HC).
Moriya 1979^216^	80 chronic SZ 80 parents of SZ (relatives) 80 HC	*‐NR‐* ^†^	‘S’ shaped figure	Free viewing	In most chronic SZ cases, the eye movements were inactive (the SZ patients tend to stick to one location). The range of movement within points of fixation for chronic SZ and their relatives was limited (for chronic SZ, more limited than that of their relatives). Fixation times of SZ patients and their parents were longer, compared to HC (fixation times of SZ patients longer than of their parents). A correlation between low eye movement and a high rating of the overall severity of symptoms was observed.
Kojima *et al*., 1990^214^	10 acute SZ 50 chronic SZ 20 remitted SZ 25 methamphetamine psychosis 21 temporal lobe epileptics with left‐sided spike focus 12 temporal lobe epileptics with right‐sided spike focus 50 HC	EEM (especially MESL and RSS)	‘S’ shaped figures	‘S’‐shaped figure procedure (retention and comparison tasks)	Short MESL can be an indicator of a chronic process of SZ. The chronic SZ patients have a significantly shorter MESL than acute SZ, remitted SZ, methamphetamine psychotics, temporal lobe epileptics with left‐sided spike focus, temporal lobe epileptics with right‐sided spike focus and HC. SZ groups have significantly lower RSS than the non‐SZ patient groups and the HC. Therefore, lowering of the RSS may be a nosologically specific indicator for SZ. A significant negative correlation between the RSS and negative symptoms (in the chronic SZ group).
Matsushima *et al*., 1992^283^	20 SZ 18 patients with frontal lobe lesions 20 HC	EEM (especially RSS)	‘S’ shaped figure	‘S’‐shaped figure procedure	Patients with right frontal lobe lesions and SZ patients have lower scores than HC for the NEFs, total TESL and MESL. The RSS is low only among patients with SZ.
Matsushima *et al*., 1998^193^	Group A and B, each comprising: 30 SZ 70 non‐SZ subjects (i.e. 10 each of patients with depression, methamphetamine psychosis, alcohol psychosis, anxiety disorder, temporal lobe epilepsy, frontal lobe lesions, and HC)	NEFs and RSS	‘S’ shaped figures	‘S’‐shaped figure procedure (retention and comparison tasks)	Obtained discriminant function: D = 10.265 − (0.065 × NEF + 0.871 × RSS) used to separate outpatients with SZ from individuals with depression, methamphetamine psychosis, anxiety disorders, temporal lobe epilepsy or frontal lobe lesions and HC. Sensitivity and specificity (in group A): 76.7% and 81.4% respectively; Sensitivity and specificity (in group B): 73.3% and 84.3% respectively.
Kojima *et al*., 2001^121^	145 SZ 116 patients with depression 124 HC	NEFs and RSS	‘S’ shaped figures	‘S’‐shaped figure procedure (retention and comparison tasks)	Using the discriminant formula from Matsushima *et al*., 1998^194^: D = 10.265 − (0.065 × NEF + 0.871 × RSS), patients with SZ could be discriminated from depressed patients and HC with a sensitivity of 89.0% and a specificity of 86.7%. The RSSs of patients with SZ are significantly lower than those of depressed patients or HC (with no significant difference existing between the RSSs for depressed patients and those for HC).
Suzuki *et al*., 2009^217^	251 SZ 389 non‐SZ (i.e. 111 patients with mood disorders 28 patients with neurotic disorders 250 HC)	TESL and RSS	‘S’ shaped figures	‘S’‐shaped figure procedure (retention and comparison tasks)	The stepwise regression analysis selected the TESL and RSS as valid parameters for discriminating between SZ and non‐SZ. Using the RSS and TESL as prediction parameters, D = 4.100 − (0.001 × TESL + 0.332 × RSS), patients with SZ could be discriminated from non‐SZ with a 73.3% sensitivity and a specificity of 79.2%.
Sprenger *et al*., 2013^60^	32 SZ 33 HC	Number, location and duration of fixations; number and amplitude of saccades; scan path length *(6 in total)*	Pictures regarding daily situations	Free viewing	Longer fixation times and fewer fixations independent of cognitive or emotional content
*(iii) Combination of eye‐movement tests*					
Arolt *et al*., 1998^117^	14 SZ (residual subtype) 17 HC	Mean gain; number of saccades; number of errors *(3 in total)*	(Exp.1.) Moving target in a triangular trajectory over a white background; (Exp.2.) Moving target in a triangular trajectory with a visual distractor (picture of the New York skyline i.e. numerous white spots on a black background); no target for voluntary saccadic task	Smooth pursuit task without (Exp.1.) and with a visual distraction (Exp.2.); Voluntary saccadic eye‐movement task	A stepwise procedure selected the three most essential parameters concerning the discrimination result, namely mean gain (%) and the number of saccades (both parameters obtained from smooth pursuit tasks), and the number of errors (obtained from the voluntary saccadic experiment). The correct classification rates were 90.3% (re‐substitution method) and 83.9% (jackknifed classification). A straightforward suggestion that not by one single task, but possibly by their combination, eye movements might serve as a diagnostic tool. Moreover, paradigms that require more substantial involvement of cognitive factors may have a more excellent heuristic value.
Benson *et al*., 2012^57^	*Training set*: 88 SZ 88 HC *Retest group*: 26 SZ 8 HC *Novel group*: 36 SZ 52 HC	Combination of machine learning models with gaze parameters (obtained from eye movement tasks)	Faces, landscapes, unfamiliar computer‐generated images (fractals)	Free viewing; smooth pursuit task; fixation stability test (with flanking distractor)	Authors used algorithms to model the eye‐movement data, including two forms of supervised machine learning, i.e. GBDTs and PNNs. The GBDT model achieved separation of the 88 SZ cases from 88 HC; its predictive validity on retest and novel groups was 87.8%. With a trained PNN model (that used data from all subjects), it was possible to discriminate SZ cases from healthy controls with 98.3%.
Miura *et al*., 2014^118^	40 SZ 69 HC	Scan path length; V‐position gain; fixation number; fixation duration; S/N ratio *(5 in total)*	Images of natural environments, buildings, everyday items, foods, faces, animals, fractal patterns; In the smooth pursuit eye movement test: target moving horizontally and on Lissajous trajectories	Free viewing test; Smooth pursuit test (fast Lissajous and horizontal pursuit paradigms); Fixation stability test (far distracter paradigm)	Canonical discriminant analysis with a stepwise procedure was conducted. 5 of the 65 eye movement variables were selected to create an integrated eye movement score (Y), which effectively represents eye‐movement abnormality. Y = 0.03 × {scan path length (free viewing)} + 2.01 × {V‐Position gain (fast Lissajous)} + 0.03 × {Fixation number (fast Lissajous)} + 0.37 × {Fixation duration (far distracter)} − 1.53 × {S/N ratio (horizontal pursuit)} − 4.92 The discriminant analysis rate was 89.9% (re‐substitution method) and 88.1% (leave‐one‐out cross‐validation method), with a sensitivity and specificity of 0.78 and 0.94, respectively.
Morita *et al*., 2017^42^	85 SZ 252 HC	Scan‐path length Horizontal position gain Fixation duration *(3 in total)*	Images of fractal patterns, foods, natural environments and animals (selected from the International Affective Pictures System), faces	Free viewing; Smooth pursuit test (fast Lissajous paradigm within); Fixation stability test (far distractor paradigm)	Eye movement measures, with the largest group differences specified by Bonferroni corrected *P*‐values, were chosen as candidates for the integrated eye movement score. The stepwise canonical discriminant analysis reduced seven measures to 3. Used discriminant function (y): y = 0.0312 × {scan path length} + 5.614 × {horizontal position gain} + 0.0000203 × {duration of fixations} − 9.637 The eye movement score correctly classified 82.5% of the participants (in the re‐substitution method) and 81.9% (in the leave‐one‐out cross‐validation method). The sensitivity and specificity of the score were reported to be 0.79 and 0.84, respectively.

^†^ *‐NR‐* Although Moriya,^216^, ^218^ did not suggest a specific eye movement discriminator, his studies pointed out atypical scanning patterns, present among chronic schizophrenia (SZ) patients.

This significantly contributed to the SZ research domain. Therefore, the Authors have included these studies on the list.

CNS, central nervous system; EEM, exploratory eye movement; ETD, eye‐tracking dysfunction; GBDT, gradient boosted decision trees; HC, health controls; MESL, mean eye scanning length; NEFs, number of eye fixations; PNN, probabilistic neural networks; RSS, responsive search score; S/N ratio, signal‐to‐noise ratio; SPEM, smooth pursuit eye movements; TESL, total eye scanning length.

In 2012, Benson *et al*. examined a large group of SZ patients and HC using a free‐viewing task (combination of pictures included e.g. luminance‐balanced natural and manmade environments showing information at different spatial scales; expressive, neutral and occluded faces; animals; and unfamiliar computer‐generated images); smooth pursuit tracking, which involved tracking a 0.5° circular target for 20 s as it moved sinusoidally on the horizontal meridian or in Lissajous patterns (in horizontal and vertical space). In addition, as a proxy for saccadic inhibition, the authors conducted steady fixation tasks, where individuals were required to maintain their gaze only on a central circular target for 5 s and to ignore a flanking distracter appearing either 1.43° or 2.86° to the left or right of the central fixation. Notably, the authors reported being able to obtain data from both groups, as all participants could complete the mentioned tasks. Extracted eye movement performance measures supported the concept of highly accurate classifier models. Data from 298 assessments were used to train a network model, which resulted in predicted classification accuracy of 98.3%.^57^ The same group highlighted that eye movement abnormalities are stable traits, independent of the neuroleptic medication and mental state at the time of testing. Furthermore, eye movement abnormalities show no correlation with the presence or absence of cigarette smoking.^57^

Scan path patterns provides valuable information when and where a subject shifts attention during visual exploration, by recording and analyzing fixation sequences.^53^ Sprenger *et al*. reported that scan path patterns differ significantly between HC and patients with SZ.^60^ In their study, seven gaze metrics parameters were determined: (i) fixations (their location, number and duration); (ii) saccades (their number and amplitudes); and (iii) the scan path length. Additionally, to test for changes over the exploration time, the exploration time of 20 s was divided into four intervals (5 s each). In line with earlier conclusions, fewer but longer fixations, as well as smaller saccades (resulting in shorter scan path length), were reported in SZ patients.^53^, ^240^, ^248^, ^269^, ^270^, ^271^, ^272^, ^273^, ^274^ Cluster analyses showed that patients fixated on fewer areas of interest (making fewer fixations per cluster) and had a longer total fixation time within each cluster. Nearly all patients reported to have had enough time to grasp the content of the presented stimuli, nevertheless, they gathered less visual information than HC. Therefore, these longer fixations may reflect a more general problem of disengaging attention. Together, the findings underline the hypothesis of a specific exploration behavior in patients that differs considerably from that in HC independently of the cognitive or emotional content of a presented image. The use of scan path similarities in discriminant analysis resulted in 85% of participants being classified correctly to either the patient or control group.

In 2014, another independent research group under Miura's lead conducted a discrimination analysis of SZ patients with a linear classifier.^118^ An integrated eye movement score, significantly different between patients with SZ and HC, was introduced that might be useful in the early diagnosis of SZ or its prodrome phase, where subjective symptoms are relatively obscure. Therefore, it might effectively assist physicians’ diagnosis. Five of the 65 eye movement variables were selected: (i) scan path length (free viewing test); (ii) position gain; (iii) the number of fixations (fast Lissajous paradigm within smooth pursuit test); (iv) S/N ratio (horizontal pursuit paradigm within smooth pursuit test); and (iv) fixation duration (far distracter paradigm within fixation stability test). The correct rate of the discriminant analysis of the selected five variables was 89.9% (re‐substitution method) and 88.1% (leave‐one‐out cross‐validation method) with a sensitivity and specificity of 0.78 and 0.94, respectively. Such accuracy was comparable or even better than the previously reported classifications (^275^, ^276^, ^277^, ^278^ cited by Miura *et al*.^118^).

In 2017, Morita *et al*. reported an integrated eye score that could distinguish patients with SZ from HC with 82% accuracy.^42^ Three of 75 eye movement variables have been selected: (i) scan path length (obtained through free viewing test); (ii) horizontal position gain (obtained through fast Lissajous paradigm within smooth pursuit test); and (iii) fixation duration (obtained through far distracter paradigm within fixation stability test). The aforementioned study again confirms the utility of gaze metrics in the identification process of SZ. The authors suggest that the use of multiple eye movement measures, to classify patients with SZ, is highly efficient and results in a discriminative accuracy of 80–90%.

Furthermore, to show eye movement abnormalities and their relationship with a patient's social/intellectual functioning,^182^, ^234^ Morita *et al*. started to actively study the association between eye movement abnormalities among patients with SZ and working hours (obtained through the Social Activity Assessment), and factors related to intelligence and education (years of education, estimated premorbid IQ and Wechsler Adult Intelligence Scale full‐scale IQ).^44^, ^182^, ^235^ Furthermore, following the findings of a present correlation between exploratory eye movements and brain structure,^221^, ^224^ Morita *et al*. extended their research. In 2019, Morita *et al*. showed that eye movement characteristics were positively correlated with cortical thickness (of the left pars opercularis) among patients with SZ.^226^ To gain more insights into social/intellectual deficits and visual abnormalities among patients with SZ and individuals in the high‐risk state for psychosis, further investigations of the relevance between exploratory eye movements, individuals’ brain structures and everyday life functioning need to be undertaken in future studies.^44^, ^231^

The aforementioned modern studies provide strong evidence that a multivariate eye movement phenotype (such as the integrated eye movement score, for example) may accurately distinguish SZ cases from HC at a rate far beyond any single gaze parameter measure. In conclusion, eye movement abnormalities appear to be stable traits of SZ. Tests that implement eye movement variables may supplement current symptom‐based diagnoses; hence, they have considerable power to discriminate schizophrenia patients from HC individuals. Analysis of scan paths might reveal differences within the psychoses not necessarily detectable by temporal measures alone. Furthermore, eye‐tracking tools are cost‐effective and can be a relief to the medical staff, as, compared with time‐consuming neuropsychological assessments carried out by highly‐qualified individuals, eye movement recordings can be performed by trained assistants. Although the currently available eye movement scores are not meant to replace criteria that rely on clinical observations and self‐reports, future studies should test the possibility of gaze metrics distinguishing SZ from other psychiatric disorders until a set of biomarkers of SZ, increasing the chance of early intervention, will raise.

## Real‐life versus laboratory experiments: Gazing toward the future

It is essential to mention that experimental paradigms influence what the scientific community accepts as truth. Dowiasch *et al*. reported that some of the results obtained from a real‐life study were contradictory to previous findings from laboratory experimental settings.^86^ More experiments comparing the gaze metrics under ecologically valid conditions, supported with wearable eye trackers, need to be undertaken. Until now, eye measurements are collected indoors through visual tests, such as free viewing, smooth pursuit and fixation stability. To better understand the interplay between cognition and vision in psychiatric disorder, introducing real‐life situations into clinical experimental paradigms that use cognitively informative paradigms, grouped under the umbrella terms of learning and decision‐making, needs to be addressed. Capturing ecologically relevant features of decision‐making, for example, may reveal cognitive deficits not apparent in ‘simpler tasks’.^151^ Following the thought that extraction task‐relevant information and choice selection (according to internal preferences and/or given experimental task) require a balance between receptor structures and the central circuit, information processing experimental paradigms may allow the clinical community to gain insight into the decision‐constructing process (through gaze metrics) and establish a comparison across various psychiatric disorders.^279^, ^280^, ^281^ Furthermore, studying decisional processes should be of great importance for SZ research, as patients commonly have motivational problems.^236^

Seemingly subtle visual impairments happen to influence real‐world functioning and impoverish an individual's life quality drastically. It is crucial to intensively replicate now‐classic paradigms, as well as design novel real‐life inspired experimental paradigms to successfully transfer gained knowledge into diagnosis protocols, characterized with high sensitivity and specificity. Therefore, combining eye movement measurements (serving as an indirect output of cognitive processes) with behavior paradigms may help the scientific community to isolate key processes that underlie psychotic disorders.^2^ As the argument over generalized impairments is clouded by the fact that until now there is no clear neuropsychological signature of SZ, information‐processing‐based investigations, supported with modern eye‐tracking devices, have the potential to bring the medical community closer to the core of SZ.^24^, ^62^

## Discussion and Summary of Evidence

Vision science is the most coherent, integrated and prosperous branch of cognitive science.^29^, ^30^, ^98^ Eye gaze metrics, probing human underlying believes, intentions, behavior and choices, spawn the field of neurocognition, where the merging of vision science and cognitively informative paradigms produces an array of scientifically recognized paradigms to study cognition. Eye movements promote understanding of how patterns of retinal activation are transformed into meaningful visual experiences, which in turn may be labeled with a specific reaction (behavior). However, while capturing mental processes of interest in cognitive psychology progresses at a relatively fast pace, the progress made in understanding the underlying mechanisms of psychiatric disorders has been surprisingly slow. In this scoping review, the authors identified journal articles published between 2010 and 2020 that addressed eye movement measurement methodology in SZ research and diagnostic field.

Smooth pursuit, free viewing and fixation stability tests offer an individual contribution to future studies of the neurobiological disturbances not only in SZ, but in other disorders.^282^ Data from multiple gaze metrics have confirmed the existence of significant differences between patients with SZ and HC.^42^, ^57^, ^112^, ^115^, ^116^, ^117^, ^118^, ^237^ Furthermore, measurements of individuals’ exploratory eye‐movements (while comparing stationary displayed ‘S’ shaped figures) provide important results. For example, in comparison with HC, patients with SZ show fewer eye fixations, longer mean duration of fixation and shorter mean scanning length (narrower range of eye movements).^121^, ^221^, ^283^ It has also been reported that the results of their parents were mid‐way between those of HC and SZ patients.^193^, ^216^ Several studies concluded that EEMs are a potential biomarker of SZ.^113^, ^115^, ^121^, ^193^, ^214^, ^284^ Importantly, among the five commonly used parameters obtained from exploratory eye movement tests, including the NEFs, mean eye scanning length, TESL, cognitive search score and RSS; only the RSS, which measures the pattern of eye fixations after the question, (‘*Are there any other differences?*’, has been pointed out to be vulnerable to SZ.^115^, ^121^, ^219^, ^224^

Furthermore, free viewing tests that involved various types of stimuli (i.e. landscapes, situations of interpersonal communication, normal and degraded faces) have shown that patients with SZ are characterized with abnormal scan path length and restricted scanning style. Thus, atypical scan path deficits and saccadic impairments are considered a trait marker of SZ.^53^, ^285^

Given that eye movement assessments are non‐invasive for patients, a promising future clinical research area is developing to evaluate potential relationships between disease characteristics and social functioning in patients with SZ.^1^, ^2^, ^20^, ^61^ The growing number of reports aiming to understand the relationship between eye movement characteristics, intellectual functioning and differences in brain structures across patients with SZ provides valuable conclusions regarding cognitive impairments and social skills deficits,^221^, ^222^, ^224^, ^225^, ^226^, ^227^, ^228^, ^229^, ^230^, ^286^ which in turn may be useful for therapeutics.^17^, ^20^, ^182^, ^225^, ^228^, ^287^, ^288^

Consequently, the next decade of SZ research is likely to witness eye movement measurements being actively used as potential biomarkers and applied to cognitively informative experimental paradigms. Future research questions will require a more profound analysis of gaze metrics during viewing tasks rather than quantification of stabilized fixations. For this reason, more complex experimental paradigms (competitive cognitive tasks embracing attention‐demanding components) should be translated from the well‐grounded neuroscientific methods and carried out to study visual exploration among individuals with SZ. Cognitively informative paradigms, which (i) demand to act on and manipulate a given problem/task/information; (ii) provide information about patients’ behavior (in laboratory and real‐life environment); and (iii) disclose patients’ visual scanning patterns, are likely to offer an individual contribution to future studies of the neurobiological disturbances involved in SZ.

Until now, clinicians cannot draw a solid line between patients with different underlying pathologies, experiencing differing types and degrees of impairment depending on the nature of given task. With this in mind, multidisciplinary contributions of cutting‐edge experimental paradigms that use eye‐tracking methodologies are required. This will require a coordinated effort of multiple scientific disciplines, including psychiatry, psychology, neuroscience and cognitive science. Such joined effort will enable us to illustrate the characteristics of one's information processes and gaze metrics patterns under various environmental conditions. Such combination will be particularly useful in gaining insights into cognitive deficits and visual abnormalities among patients with different dimensions of SZ and individuals in the high‐risk state for psychosis.

## Limitations

Our scoping review is likely generalizable to journal articles and systematic reviews acquired through the Kyushu University Open Access Policy.

## Disclosure statement

The authors have no conflicts of interest to declare.

## Author contributions

Y.H. encouraged A.W. to investigate eye movement abnormalities in patients with schizophrenia and supervised this scoping review's findings. A.W. wrote the manuscript. K.U. and Y.H. revised the manuscript. All authors contributed to and approved the final manuscript.
